# Macrophage Biocompatibility of CoCr Wear Particles Produced under Polarization in Hyaluronic Acid Aqueous Solution

**DOI:** 10.3390/ma11050756

**Published:** 2018-05-08

**Authors:** Blanca Teresa Perez-Maceda, María Encarnación López-Fernández, Iván Díaz, Aaron Kavanaugh, Fabrizio Billi, María Lorenza Escudero, María Cristina García-Alonso, Rosa María Lozano

**Affiliations:** 1 Cell-Biomaterial Recognition Lab., Department of Cellular and Molecular Biology, Centro de Investigaciones Biológicas (CIB-CSIC), Ramiro de Maeztu 9, 28040 Madrid, Spain; bpm@cib.csic.es (B.T.P.-M.); lfmarien@gmail.com (M.E.L.-F.); 2 Department of Surface Engineering, Corrosion and Durability, Centro Nacional de Investigaciones Metalúrgicas (CENIM-CSIC), Avda. Gregorio del Amo 8, 28040 Madrid, Spain; ivan.diaz@cenim.csic.es (I.D.); escudero@cenim.csic.es (M.L.E.); crisga@cenim.csic.es (M.C.G.-A.); 3 Department of Orthopaedic Surgery, David Geffen School of Medicine, University of California Los Angeles, Orthopaedic Hospital Research Center, 615 Charles E. Young Dr. South, Room 450A, Los Angeles, CA 90095, USA; akavanaugh@mednet.ucla.edu (A.K.); fabrizio.billi@gmail.com (F.B.)

**Keywords:** polarization, CoCr alloy, wear particles, hyaluronic acid, macrophages biocompatibility

## Abstract

Macrophages are the main cells involved in inflammatory processes and in the primary response to debris derived from wear of implanted CoCr alloys. The biocompatibility of wear particles from a high carbon CoCr alloy produced under polarization in hyaluronic acid (HA) aqueous solution was evaluated in J774A.1 mouse macrophages cultures. Polarization was applied to mimic the electrical interactions observed in living tissues. Wear tests were performed in a pin-on-disk tribometer integrating an electrochemical cell in phosphate buffer solution (PBS) and in PBS supplemented with 3 g/L HA, an average concentration that is generally found in synovial fluid, used as lubricant solution. Wear particles produced in 3 g/L HA solution showed a higher biocompatibility in J774A.1 macrophages in comparison to those elicited by particles obtained in PBS. A considerable enhancement in macrophages biocompatibility in the presence of 3 g/L of HA was further observed by the application of polarization at potentials having current densities typical of injured tissues suggesting that polarization produces an effect on the surface of the metallic material that leads to the production of wear particles that seem to be macrophage-biocompatible and less cytotoxic. The results showed the convenience of considering the influence of the electric interactions in the chemical composition of debris detached from metallic surfaces under wear corrosion to get a better understanding of the biological effects caused by the wear products.

## 1. Introduction

Macrophages are cells involved in inflammatory processes [1]. All orthopedic biomaterials may induce a biologic host response to generated wear debris, which is strictly dependent on the nature of the debris. Metal wear particles and metal ions from prosthetic devices may induce a cascade of adverse cellular reactions that may include inflammatory complications, macrophage activation, bone resorption, and, although rarely, neoplasia [2,3]. In this context, macrophages play a decisive role in the hostile inflammatory reactions that can lead to implant loosening and failure.

Implanted metal surfaces in biological environments are exposed to cells and to physiological milieu interacting between them, an interaction that affects both the cells and the metallic surface. Implanted metallic materials, such as CoCr alloys, undergo dissolution and formation of a passive film that is affected by factors such as pH, ions present in the physiological medium, temperature, and biopotentials. Biopotentials are natural electrical properties that control the normal growth and development of different types of cells and tissues [4,5]. When a tissue is injured, its potentials undergo alterations to the normal potential of intact tissue [6,7]. Both biopotentials and injury potentials are found in bone and these potentials induced between injured and intact tissues persist until the tissue heals. Potentials in injured tissue can span over hundreds of microns and are generated by electric fields or ions flowing through the injured tissue [8,9] with a range of 10–100 mV/cm [10]. Assuming the resistivity of soft tissues to be 100 Ω cm [9,11], the resulting current density is in the 1–100 μA/cm^2^ range [8,12]. Fukada and Yasuda had already described in 1957 the piezoelectric nature of the bone tissue [13]. Endogenous electrical properties of bone may play a role in the feedback mechanism of bone remodeling and development [14,15]. In vivo, these electrical signals work in collaboration to provide the correct environment for normal bone growth and development, but can be disrupted or altered by an injury after a trauma and during the healing process. Moreover, the resulting voltage gradients may induce modifications in the electrochemical potential of metallic implants and consequently may affect their surface properties.

Díaz et al. [16] recently characterized the CoCr alloy oxide films in a phosphate buffer solution containing 3 g/L of hyaluronic acid, the approximate concentration found in the synovial fluid of healthy joints [17], and under potentials with current density similar to those reported for injured tissues (1–100 μA/cm^2^). Potentiostatic pulses applied during the growth of the CoCr oxide film produced a modification of the film that affected its chemical composition, thickness, and structure compared to the passive film formed in air [16]. These modifications induced surface heterogeneities at the atomic scale, geometric irregularities, such as nano-roughness, and a variation of the oxide composition [16]. Moreover, application of potentials of 0.7 V vs. Ag/AgCl induced changes in the oxide layer with the formation of 10–50 nm diameter nanopores, uniformly distributed along the surface and an increase in Cr (VI) and Mo (VI) concentration [16].

Despite the presence of the passive film, metals are susceptible to corrosion, particularly in aqueous environments, which may affect the surrounding tissue. Corrosion events generate electrical currents due to electron transfer from ions in the solution to the metallic surface where reactions are occurring. Wear-corrosion phenomena and micromotion or fretting-corrosion mechanically removes material, including the passive film, causing continuous activation/repassivation cycles [18]. These continuous and dynamic processes not only weaken the surface performance but also lead to an increase in the debris around the implant. Wear debris is considered one of the main factors responsible for aseptic loosening of orthopedic endoprostheses [19,20]. Implant failure due to aseptic loosening, or osteolysis, may result from the release of wear debris or electrochemical ions generated during corrosion events [20,21,22].

From the electrochemical point of view, on the metallic surfaces of implants, the breakdown of the passive film under the wear-corrosion process causes a drastic decrease in the open circuit potential of the metal towards negative potentials, i.e., from the passive to active state. This situation can suppose a polarization of about 500–700 mV with respect to the original open circuit potential. The change from the passive to active state can be induced mechanically under wear and electrochemically applying anodic polarization on the tribological system. Several researchers have studied the wear corrosion processes by application of anodic potentiodynamic polarization under wear processes [23,24].

The object of this paper was to evaluate the biocompatibility of particles produced during wear-corrosion assays of a CoCr alloy at potentiodynamic range to cover a wide polarization window on the samples. The hyaluronic acid, the lubricant component of the synovial liquid, was selected as the electrolyte for the generation of wear particles in conditions that represent more closely the prosthesis environment. Since macrophages are the main cells involved in the primary response to foreign bodies, cytotoxicity and biocompatibility of the wear particles were evaluated using these cells, measuring lactate dehydrogenase and mitochondrial activity, respectively.

## 2. Results and Discussion

### 2.1. Wear-Corrosion Tests

The interaction of physiological fluids with the bearing surfaces of hip implants is of great importance in the research of artificial joint lubrication, although this study has been so far little explored.

**Figure 1:**
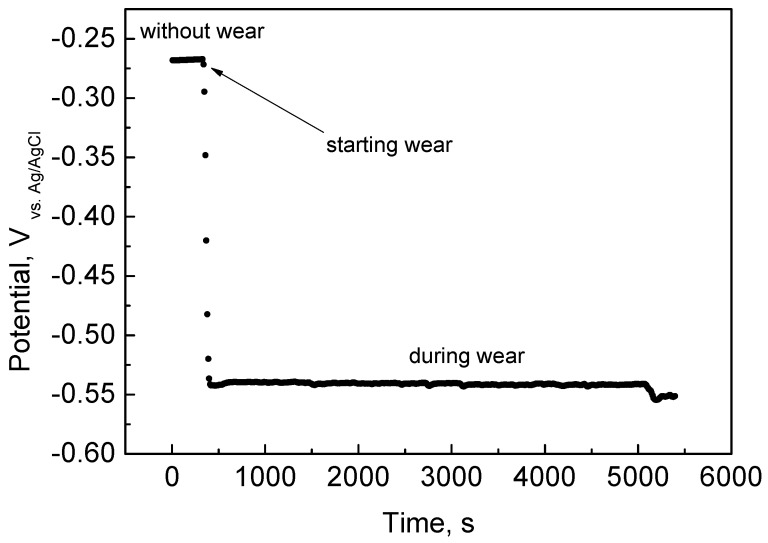
Open circuit potential of HCCoCr disks under wear. Measurement of the open circuit potential before and during the wear corrosion test of HCCoCr in PBS containing 3 g/L HA (PBS-HA).

The effect of sliding of the alumina ball on the HCCoCr alloys is clearly shown in the drastic change in the open circuit potential. As an example, Figure 1 shows the change in the open circuit potential when the HCCoCr surfaces in PBS supplemented with 3 g/L HA (PBS-HA) are subjected to wear. The open circuit potential without wear was around −0.25 V versus Ag/AgCl, decreasing sharply when the alumina ball (pin) started the circular movement under 5 N load at 120 rpm. At this moment, the open circuit potential decreased until achieving values of about −0.55 V vs. Ag/AgCl, i.e., about 300 mV, and remained constant until the end of the test. The reduction in the potential value towards more negative values indicates that the HCCoCr surface becomes electrochemically active. This variation is due to the breakdown of the passive film under sliding, promoting the release of metallic ions and particles.

**Figure 2:**
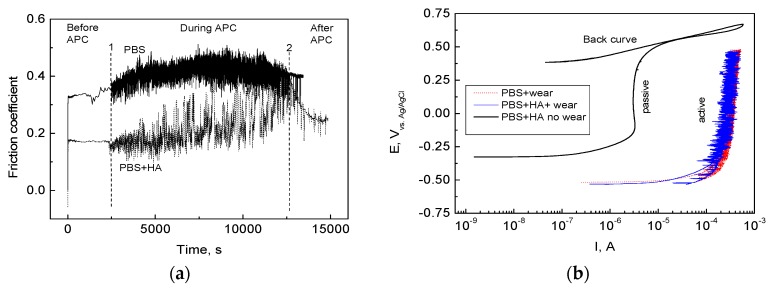
Friction coefficient of HCCoCr/alumina pair (**a**) and anodic polarization curves (APC) of HCCoCr disks (**b**) during wear corrosion tests in PBS and PBS containing 3 g/L HA (PBS-HA). Measurement of the friction coefficient before, during, and after application of anodic polarization current (APC). Anodic polarization curve for HCCoCr alloy in PBS-HA without wear is added for comparative analysis.

**Figure 3:**
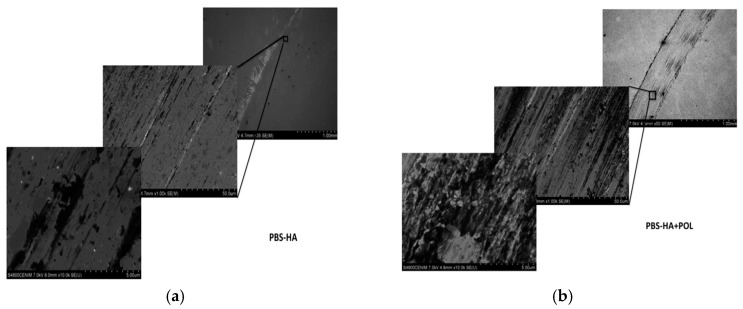
Secondary electron images of wear tracks on HCCoCr disks. Images by SEM of HCCoCr samples in PBS-HA under wear: (**a**) at the open circuit potential (PBS-HA) and (**b**) applying anodic potentiodynamic polarization (PBS-HA+POL).

Figure 2 panels a and b show the coefficient of friction (COF) for HCCoCr/alumina pair in PBS and PBS supplemented with 3 g/L HA (PBS-HA) during anodic potentiodynamic polarization and the anodic polarization curves drawn at 10 mV/min of HCCoCr in PBS and PBS containing 3 g/L HA under wear conditions (between point 1 and 2 in Figure 2a), respectively. The anodic polarization curve of HCCoCr in PBS-HA without wear has been also added in Figure 2b for comparative reasons. It can be seen that under sliding at the corrosion potential (before point 1 in Figure 2a), the COF was significantly higher in PBS than in PBS-HA. This result agrees with the hypothesis that the hyaluronic acid has a known lubricant role in the joint, acting as a shock absorber [25] and thus facilitating smooth joint movement by reducing friction between both surfaces. At the next stage (from point 1 to 2, in Figure 2a), the difference between both COF (in PBS and PBS-HA) remained, but higher fluctuations were detected. The fluctuations could be related to the continuous formation of hard particulate matter that enhances friction between both counterparts and decreases the friction when ejected from the track to surrounding areas where it accumulates (Figure 3). The load applied on the CoCr surfaces while sliding activates mechano-chemical reactions, causing not only the detachment of the passive film [26] but also bulk material resulting in an increase of COF. The hyaluronic acid in PBS maintains the lubricant effect during most of the wear corrosion tests.

As a consequence of mechanically assisted corrosion, the passive film on the HCCoCr surface was rapidly broken in both media, PBS and PBS-HA, producing an increase of approximately 3 orders of magnitude in current (Figure 2b) with respect to the anodic polarization curve without wear. Corrosion progresses on the wear track drawn by the sliding of alumina ball on the HCCoCr disks (Figure 3). Having in mind the wide passive region seen in the anodic polarization curve drawn without wear (Figure 2), the potential applied could be employed in forming rapidly the new oxide film. However, the sliding rate is quick enough to avoid the repassivation and formation of new protective chromium oxides. The constant value of the current density around 1 mA (three orders of magnitude higher than without wear) indicates that under these experimental conditions (5 N load and sliding rate of 120 rpm), the passive film is destroyed and remains in an active state until the end of the test.

**Figure 4:**
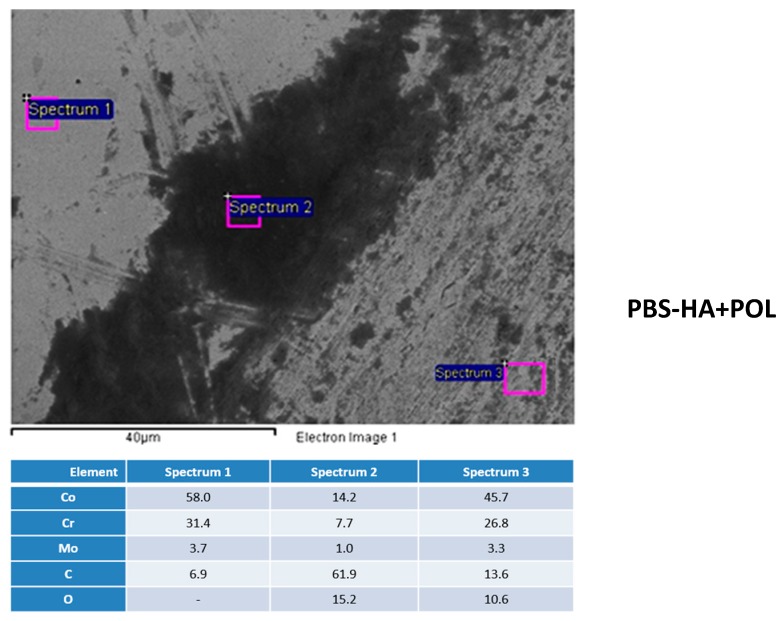
HCCoCr surface after wear corrosion applying anodic potentiodynamic polarization. Secondary electron image and EDS analyses away from the track, in the immediate vicinity, and inside the track in the HCCoCr surface in PBS-HA+POL.

Figure 3 shows the secondary electron (SE) images of the tracks of HCCoCr in PBS-HA after wear corrosion tests, at the corrosion potential (PBS-HA) and under anodic potentiodynamic polarization (PBS-HA+POL). In both cases (a) and (b), debris is accumulated in the immediate vicinity of the wear tracks, but the surface inside the track is especially altered when anodic potentiodynamic potential is applied. Figure 4 shows, as an example, the semiquantitative analysis taken by EDS of the three areas of interest in the HCCoCr alloy immersed in PBS-HA after wear corrosion under polarization (PBS-HA-POL): away from the track (spectrum 1), immediate vicinity (spectrum 2), and inside the track (spectrum 3). The most important feature found is the high % C content accumulated in the vicinity of the track. It means that the debris is mainly composed of C and O, the greatest proportion probably coming from the hyaluronic acid.

**Figure 5:**
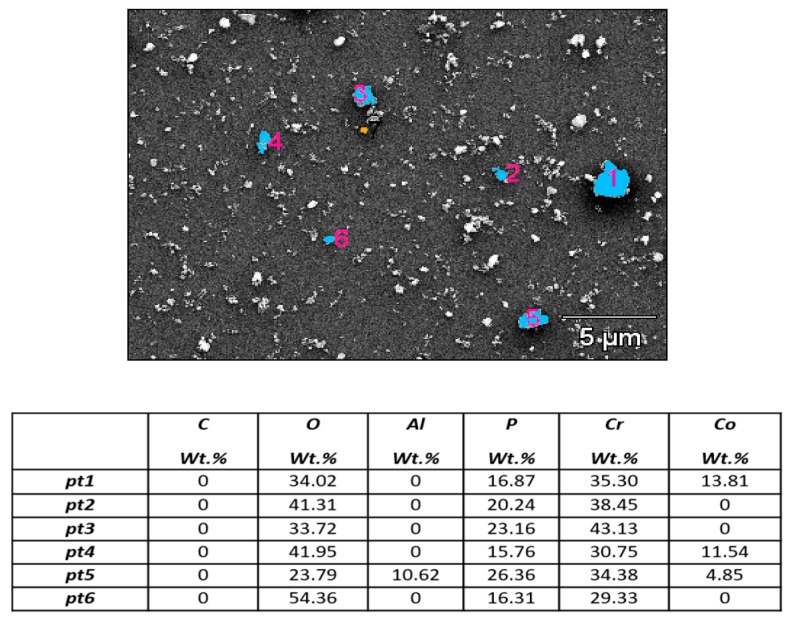
Secondary electron images of wear particles. Particles were collected from wear corrosion tests performed in PBS containing 3 g/L HA (PBS-HA) and deposited on silicon wafer to analyze the chemical composition of detached particles. Blue colors represent the particle where EDS has been performed and the particle number shown in pink is correlated to the pt shown in the table attached.

The morphology and chemical characterization of the wear particles detached during wear corrosion tests revealed some interesting results. Figure 5 shows, as an example, the secondary electron image of wear particles collected from the tribocorrosion test in PBS containing 3 g/L HA and the semiquantitative analysis of some particles, identified from 1 to 6 and marked in blue color.

The statistical results of the effect of the corrosive medium and polarization applied in the wear corrosion tests on the chemical composition of the wear-detached particles collected appear in Table 1. In this table, three condition numbers assigned to 1, the PBS corrosive medium, 2, the PBS-HA corrosive medium without applying polarization, and 3, the PBS-HA corrosive medium applying polarization (PBS-HA-POL), have been considered. Mean, standard deviation, minimum and maximum value, C25 and C75, and median are shown.

It can be seen that the particles are mainly composed of Co, Cr, Mo, P, C and O, with some traces of Al in some isolated particles. The Kruskal–Wallis test indicated that there are significant differences in the levels of Cr, Co, Mo, O and C, comparing the different conditions, i.e., depending on the composition of corrosive medium (PBS-condition 1 or PBS-HA-condition 2) and the application of polarization in wear corrosion tests (PBS-HA-POL, condition 3, and PBS-HA, condition 2). However, no significant differences in P and Al levels were obtained.

The results of the post hoc Mann–Whitney test used to determine which pairs differed among them are shown in Table 2. Cr levels are significantly higher in condition number 3 than 2 (*p* = 0.021). Co and Mo levels are significantly higher in condition number 3 than 1 and 2 (*p* = 0.002 and *p* = 0.001, *p* = 0.025 and *p* = 0.002, respectively). O levels are significantly lower in condition number 3 than 1 and 2 (*p* = 0.002 in both cases). C levels are significantly lower in condition number 3 than 2 (*p* = 0.017).

In summary, the statistical analysis confirmed that factors such as “composition of the corrosive medium” and “polarization applied” have an influence on the dependent variable chemical composition of the particles that is discussed immediately below.

The main significant effect of the addition of hyaluronic acid in the PBS to the wear particles detached is observed in the increase of the C content in the chemical composition of the particles. In both media (PBS, condition 1, and PBS-HA, condition 2), particles are mainly composed of Cr and O, followed by P and some Co. This chemical composition can be directly linked to the detachment of the native passive film during the wear corrosion test.

It has been proven by XPS (data not shown) that the immersion of the HCCoCr surfaces in PBS-HA causes a decrease in the Co species in the passive film and the enrichment in chromium oxide where phosphorus is included. It has been reported in literature that phosphate is adsorbed upon freshly exposed metal at the same time that ions are released into the solution until the passive layer is formed, whose composition varies significantly depending upon the environment [27]. Lewis et al. established that the corrosion, especially when associated with mechanical wear, is controlled by phosphate anions that absorb or react with the Co and Cr dissolution products. This promotes the formation of a mixed composition of phosphates, hydroxides, and oxides originating from the bulk metal.

This means that most of the particles collected after the wear corrosion tests in PBS and PBS-HA come from the native passive film (whose thickness is about 5–7 nm) and are mainly composed of chromium oxide and phosphate.

With respect to applying polarization during the wear corrosion tests in PBS-HA (condition 3), this factor has an important effect on the chemical composition of the wear particles detached. In this condition, particles are mainly composed of Cr and Co, followed by O, P, and Mo. The main significant effect of the polarization is the significant enrichment in Co, Cr, and Mo in the chemical composition of the detached particles. In this case, the wear particles produced under anodic polarization increased the Co/Cr ratio (with a value of 0.9 in comparison with a value of 0.3 found in PBS-HA without polarization). As wear particles obtained without polarization, these particles also contained P, although in a low proportion (Table 1). It has been reported in the literature [16] that the potential applied on the HCCoCr induces a change in the chemical composition of the passive film. Díaz et al. established that the increase in polarization (from 0.5 to 0.7 V) induced the preferential dissolution of cobalt whereas chromium was concentrated in the surface oxide film [16]. The passive film grown at a potential of 0.5 V vs. Ag/AgCl (into the passive region of the anodic polarization curve) consisted predominantly of Cr_2_O_3_ and Cr(OH)_3_. However, the oxidation at a potential of 0.7 V vs. Ag/AgCl caused the appearance of Cr (VI) in the passive film but Co was not increased. In the case of wear corrosion under anodic potentiodynamic polarization, the continuous sliding of the alumina ball on the HCCoCr surface did not allow the regeneration of the oxide film. Instead, an active state stimulated by polarization was induced on the surface where bulk material was directly exposed and detached to the electrolyte. Considering this situation, the results reveal that the anodic polarization on CoCr surfaces under wear-corrosion processes accelerated and induced the release of larger metallic particles with higher Co content coming from the base material.

### 2.2. Macrophage Cell Response

Macrophages are a primary immune cell type and the main cellular type involved in inflammatory processes [1] and in host response [28], so their biologic host response to wear particles generated from the implanted materials is of great interest.

Macrophage response to wear particles derived from the tribocorrosion assays was evaluated by measuring the effect on cell toxicity and respiratory activity.

**Figure 6:**
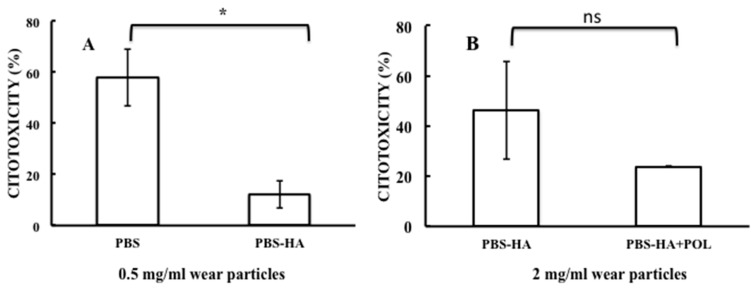
Macrophage cytotoxicity, measured as LDH activity, of cell cultures exposed for 72 h to HCCoCr wear particles. Panel A: Exposure of macrophages culture to 0.5 mg/mL wear particles. Particles were obtained in PBS and in PBS containing 3 g/L HA (PBS-HA). A *p* value of ≤0.05 was considered significant (*); Panel B: Exposure of macrophages culture to 2 mg/mL wear particles obtained in PBS containing 3 g/L HA with and without polarization application, PBS-HA+POL and PBS-HA, respectively. Experimental data were done as independent triplicate. Differences between data analyzed here were not significantly different (labeled as ns).

Cytotoxicity induced by HCCoCr wear particles was analyzed by measuring LDH activity released from cells (Figure 6), whose levels increase upon plasma membrane damage, a sign of cell death [29]. As is shown in Figure 6, exposure of macrophages cultures to wear particles induced a degree of cytotoxicity that was mainly dependent on the conditions used during wear-corrosion assays and particle concentration. As shown in Figure 6 panel A, particles concentration of 0.5 mg/mL obtained in PBS produced almost 58% cytotoxicity, a percentage that was significantly reduced to almost 12% when wear particles were generated from tribocorrosion tests in the presence of 3 g/L of hyaluronic acid (PBS-HA), an effect that could indicate a protective role of the hyaluronic acid on the metallic surface under wear stress conditions (Table 3). Concentrations of 1 mg/mL of wear particles from the PBS test produced an increase in the macrophage cytotoxicity to almost 75%, a value elevated in comparison with the cytotoxicity induced by the wear particles obtained in PBS containing 3 g/L of HA, where cytotoxicity reached 14% (data not shown). No additional increase in the cytotoxicity was observed at higher concentrations of wear particles (2 mg/mL) generated in PBS as macrophages cytotoxicity appeared comparable to the one elicited by exposure to lower concentrations of particles (0.5 and 1 mg/mL), where approximately a 64% cytotoxicity was detected (data not shown).

**Figure 7:**
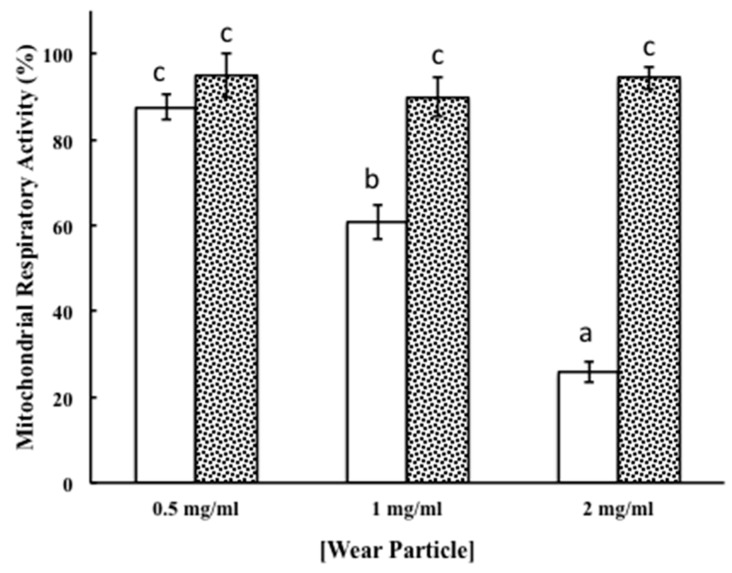
Mitochondrial respiratory activity of macrophages cell cultures exposed for 72 h to different doses of HCCoCr wear particles. Wear particles were obtained in PBS containing 3 g/L HA without (white bars) and with polarization (dotted black bars). Cell cultures were exposed to the following wear particles concentrations: 0.5, 1 and 2 mg/mL. Experiments were done as independent triplicate. Bars labeled with different letters show statistically significant differences and bars labeled with the same letter (c) show nonsignificant differences.

Particles produced in PBS containing 3 g/L of HA at concentrations of 2 mg/mL elicited an increase in the macrophages cytotoxicity that reached almost 46% (Figure 6, panel B, PBS-HA). Although such an increase was higher than the one produced by particles concentrations of 0.5 and 1 mg/mL, which were 12% and 14%, respectively, it was reduced to 24% when polarization conditions characteristic of damaged tissue were applied (Figure 6, panel B, PBS-HA+POL). Although the statistical analysis of the data from Figure 6 panel B (Table 3) gave no significant differences between results analyzed here, the application of anodic polarization to HA aqueous solution seems to have important observable differences on the mean value of the cytotoxicity. This feature could be relevant and, for this reason, verification by other biocompatibility assays is required. With this purpose, the wear particles collected from the tribocorrosion assays of CoCr alloy in PBS-HA without and applying anodic polarization were tested on macrophages cultures by measuring the mitochondrial activity. It is well known that the mitochondrial activity measurement is directly proportional to the number of metabolically active cells in culture [29] constituting a measure of cell viability and biocompatibility. As it is shown with Figure 7 by white bars, wear particles collected in the PBS-HA produced a gradual and significant reduction in the mitochondrial respiratory response of macrophages. This result seemed to be directly related to the concentration of particles to which macrophages were exposed (Table 4). Nevertheless, no reduction in the mitochondrial respiratory activity was observed in macrophages exposed to wear particles generated when polarization was applied during wear-corrosion tests. No significant effects in respiratory activity were observed in the range of particles concentrations tested (Figure 7, black dotted bars, and Table 5). The results suggest that the polarization conditions in the wear-corrosion assays in PBS containing HA at the approximate concentration found in synovial fluid seem to be beneficial to macrophage viability and biocompatibility.

The dose-dependence effect on mitochondrial respiratory activity by particles detached in PBS-HA could be explained by the chemical composition of wear particles collected from wear-corrosion tests in this solution as a decrease in Co was observed, as well as an enrichment in chromium oxide, a compound with high toxicity [30] in comparison with the composition of PBS-HA-POL wear particles. The results suggest that polarization conditions applied to an HA aqueous solution at the approximate concentration found in synovial fluid produce changes in material tribocorrosion behavior inducing wear particles that seem to be beneficial to macrophage viability and biocompatibility. Data that could explain the higher biocompatibility of wear particles generated in PBS-HA+POL could be related to the fact that under these conditions, wear processes did not allow the regeneration of the oxide film. This event could determine the creation of an active state where CoCr base material was directly exposed to the electrolyte without enough time to build up the new oxide film that induced the release of metallic particles with higher Co content probably coming from the base material.

## 3. Materials and Methods

### 3.1. Material

A high carbon CoCr alloy (hereafter HCCoCr) that complies with ASTM F75 standard was used as material. HCCoCr composition is shown in Table 6. “Double heat-treated” disks, i.e., solution treatment (ST) followed by hot isostatically pressing (HIP), of 38 mm in diameter and 4 mm thickness, were obtained from BIOMET Spain Orthopaedic (Valencia, Spain). The sample preparation consisted of grinding on SiC paper, followed by mechanical polishing with 3 µm diamond paste.

### 3.2. Wear-Corrosion Tests under Electrochemical Control

Wear-corrosion experiments were carried out on a pin-on-disk tribometer, and 6-mm diameter alumina ball pins were used as HCCoCr disk counterpart. The HCCoCr disks were 38 mm in diameter and 4 mm thick. Both disks and pins were previously washed with double distilled water and cleaned in an ultrasonic ethanol bath for 10 min. The alumina pins were placed in a pin plastic holder and fixed on the load cell. A low normal load of 5 N was applied on the counterpart. The working electrode motion was provided by a rotating motor at a rotation rate of 120 rpm that produced, at the end of the alumina ball, a circular wear track (5 mm in diameter) on the HCCoCr disk surface.

The tribometer configuration consisted of an integrated electrochemical cell (3-electrode cell) including the HCCoCr disk as working electrode, a ring-shaped Pt wire counter electrode and a saturated Ag/AgCl reference electrode. All the potentials of the HCCoCr disks during the wear corrosion tests were measured versus the reference electrode. Wear-corrosion tests were performed in Phosphate Buffer Solution (PBS) containing the following composition: 0.2 g/L KCl, 0.2 g/L KH_2_PO_4_, 8 g/L NaCl, and 1.150 g/L Na_2_HPO_4_ (anhydrous) and this PBS solution was supplemented with 3 g/L hyaluronic acid, the approximate concentration reported for the synovial fluid of healthy joints [17].

**Figure 8:**
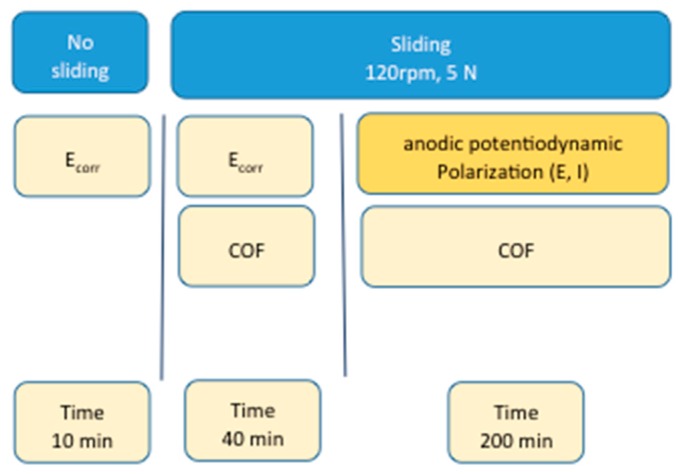
Schema of the experimental procedure of the wear-corrosion tests.

The wear-corrosion behavior was studied simultaneously measuring the friction coefficient and electrochemical parameters. The wear-corrosion tests were performed as follows (Figure 8): (a) before wear (no sliding) by the measurement of the corrosion potential for 10 min and (b) under sliding (at 120 rpm and 5 N load) in two different ways: one where a simultaneous measurement of the corrosion potential and the coefficient of friction (COF) were performed for 40 min without applying polarization and the other, applying anodic potentiodynamic polarization and simultaneous measurement of current and coefficient of friction (COF) for 200 min. The anodic potentiodynamic polarization was applied from the corrosion potential to a polarization of 1 V at a scanning rate of 10 mV/min, and back curve was drawn until reaching the corrosion potential. The back curve was also measured to analyze the repassivation ability of the HCCoCr alloy. For comparative purposes, the anodic potentiodynamic polarization without wear in PBS and PBS-HA was also measured. All experiments were carried out in triplicate.

Surface characterization of the worn surface after wear-corrosion tests with polarization and without polarization was performed by profilometry and using a JEOL-6500F microscope equipped with a field Emission Gun (FEG) coupled to an Energy Dispersive X-ray (EDS) spectrometer. Secondary electron (SE) images were taken at 7.5 keV and EDS analysis was performed at 20 keV.

### 3.3. Isolation and Characterization of Particles

Debris from tribocorrosion tests performed in PBS, PBS containing 3 g/L hyaluronic acid (both without applying polarization), and in PBS supplemented with hyaluronic acid under anodic polarization was collected for the subsequent characterization. Metallic particles were isolated, purified, and characterized following the protocol developed by Billi et al. for metal particles [31]. This procedure allows exhaustive removal of organic and inorganic impurities from the metallic particles. To completely digest the hyaluronic acid, metal particles in PBS supplemented with 3 g/L HA were digested adapting the protocol developed by Kavanaugh et al. [32].

Wear-corrosion media (PBS and the digested hyaluronic acid solutions) containing metallic particles were rotated at 28 rpm in an orbital agitator for 24 h at room temperature to disperse the metal particles evenly before their isolation. The particles were then purified via density gradient centrifugation varying from 4446× *g* to 284,000× *g* (Beckman Optima L80 XP; Beckman Instruments, Fullerton, CA,USA) through multiple layers of denaturants and metal-selective high-density layers as was described [31,32]. This led to well-dispersed particles deposited onto a 5 mm × 5 mm featureless display silicon wafer (Ted Pella, Inc., Redding, CA, USA) coated with a monolayer of marine mussel glue (Cell-TakTM; BD Biosciences, San Jose, CA, USA). The silicon wafer was then coated with 10 Å iridium [31,32].

The morphology of metallic particles was studied with a field emission scanning electron microscope (FE-SEM) (Supra VP-40; Zeiss, Peabody, MA, USA) at a voltage of 15 kV and chemically analyzed by means of Energy-dispersive spectroscopy (EDS) analysis (Thermo Ultradry feature sizing system; Thermo Electron Scientific Instruments, Madison, WI, USA).

### 3.4. Macrophages Cell Cultures Assays

The biocompatibility of wear particles was tested in a mouse macrophage cell line (J774A.1) from DSMZ Human and Animal Cell Bank. Macrophages cell cultures were exposed to different concentrations of wear particles.

Wear particles obtained from the wear-corrosion tests were centrifuged and the particle pellet was weighted, UV sterilized for 15 min, and resuspended in sterile bidistillated water and maintained in aliquots at −20 °C until use. Wear particles, just before cell cultures assays, were thawed, resuspended by vigorously mixing with a vortex, and diluted at a concentration of 20 mg/mL in Dulbecco’s Modified Eagle Medium (DMEM 41966; Gibco, BRL, Invitrogen, Thermofisher scientific, Paisley, UK) supplemented with 10% heat-inactivated fetal bovine serum (FBS; Gibco, BRL) and with a mixture of antibiotics (penicillin at 100 units/mL and streptomycin at 100 g/mL, Gibco, BRL), named as complete cell culture medium. A concentration of 20 mg/mL was used as stock solutions for the particles concentration tested in different cell assays. To assure a polydisperse distribution of the particles vigorous vortexing was applied in all experimental steps that required particles manipulation.

To evaluate the effect of HCCoCr particles on cell cultures, macrophages were seeded on 96-well culture plates at 75,000 cells/mL cell density in complete cell culture medium. A final volume of 100 μL of cell suspension in complete cell culture medium was added to each well of the 96-well plates. After 24 h in culture, cell media were removed and replaced by 100 μL of fresh complete cell culture medium containing the following concentrations of HCCoCr particles: 0, 0.5, 1 and 2 mg/mL. Cell cultures were maintained for 72 h in a cell culture chamber at 37 °C and 5% CO_2_. Incubation time was selected based on the set-up of cell cultures assays for metallic particles studies carried out in the lab and is the most commonly used time point for cell viability studies [33]. Mitochondrial activity (WST-1 assay) and plasma membrane damage (LDH assay) were used to evaluate the biocompatibility and cytotoxicity, respectively, as described below [29].

### 3.5. Mitochondrial Activity Measurement

Reduction of the WST-1 reagent (4-[3-4-iodophenyl)-2-(4-nitro-phenyl)-2H-5-tetrazolio]-1,3-benzene disulfonate (Roche Diagnostics GmbH, Mannheim, Germany)) was used to evaluate the effect of different concentrations of the HCCoCr wear particles on mitochondrial activity of macrophages cultures. The mitochondrial activity measurement is directly proportional to the number of metabolically active cells in culture. After 72 h in culture, 10 μL of the cell proliferation kit reagent WST-1 was added to each well containing 100 μL of fresh complete cell culture medium, and the mixture was incubated inside the cell culture incubator for 30 min. After incubation, 100 μL of each reaction mixture were transferred to a 96-well cell plate, and the absorbance of the samples was measured as differential absorbance, 415 nm minus 655 nm, in an iMark microplate absorbance reader (Bio-Rad, Hercules, CA, USA), using the absorbance given by complete cell culture medium as a blank. All experiments were carried out as independent triplicate.

### 3.6. Measurement of Lactate Dehydrogenase Activity

To measure and quantify the effect of HCCoCr wear particles on cell death and cell lysis, lactate dehydrogenase (LDH) activity was measured in the supernatants of cell cultures by an enzymatic assay using the Cytotoxicity Detection Kit^plus^ (Roche Diagnostics GmbH, Mannheim, Germany). Supernatants were collected from cell culture after being exposed for 72 h to different HCCoCr particles concentrations and were centrifuged for 5 min at 1024× *g*. The enzymatic assays were performed according to the LDH kit protocol provided by Roche Diagnostics (Mannheim, Germany). Complete cell culture medium was used as a control for absorbance baseline. LDH activity was measured based on differential absorbance, 490 nm minus 655 nm, in an iMark microplate absorbance reader (Bio-Rad, Hercules, CA, USA). LDH catalyzes the conversion of lactate to pyruvate, reducing NAD^+^ to NADH/H^+^, which is used by the catalyst to reduce a tetrazolium salt to a formazan salt, which is responsible for the change in absorbance at 490 nm. Quantification of LDH activity is used as an indicator of plasma membrane damage, as is a stable cytoplasmic enzyme present in all cells and rapidly release into the cell culture supernatant when the plasma membrane is damaged being a sign of cell death. The percentage of cytotoxicity is calculated taking as control a total cell lysate in the absence of any particles. The percentage cytotoxicity is calculated as described in the LDH kit protocol provided by Roche Diagnostics: Cytotoxicity (%) = [(exp. value − low control)/(high control − low control)] × 100; where experimental value (exp. value) corresponds to the absorbance of the treated sample in the study exposed to wear HCCoCr particles, low control is the absorbance from the untreated cell cultures with no particles that corresponds to spontaneous LDH released, and high control is the absorbance value obtained after total cell cultures lysis that corresponds to the maximum releasable LDH activity. The background absorbance corresponding to complete cell culture media was subtracted from the absorbance of all samples before cytotoxicity calculations. All experiments were carried out as independent triplicate.

### 3.7. Statistical Analysis of Data

#### 3.7.1. Wear Particles Analysis Data

The experimental design used to determine the effect of two factors as the corrosive medium and the application of polarization on the dependent variable, that is, the chemical composition of the particles, was a 2^2^ factorial design. In order to explain significant interaction, simple effects of one factor on the dependent variable at each single level of the other factor were computed. After this, simple effect pairwise comparisons were performed to detect levels of the second factor in which simple effects of the first factor on the dependent variable were significantly different. Kruskal–Wallis [34] and Mann–Whitney [35] nonparametric tests were used to confirm the ANOVA results. A *p*-value < 0.05 was considered as significant. All the statistical analyses were performed with the Minitab^®^ 17.1.0 software (Minitab Inc., State College, PA, USA) [36].

#### 3.7.2. Biocompatibility Analysis Data

Mean differences on cytotoxicity effects between wear particles obtained in PBS (0.5 mg/mL) versus particles in PBS containing 3 g/L of hyaluronic acid (0.5 mg/mL) and between wear particles obtained in PBS containing 3 g/L HA (PBS-HA; 2 mg/mL) without versus with polarization application (PBS-HA+POL; 2 mg/mL) were studied with Student’s *t* tests (α = 0.05), respectively.

The effects of the particles, the concentration, and their interaction on the changes in mitochondrial respiratory activity of macrophages were analyzed with a two-way analysis of variance. A *p* value of ≤0.05 was considered significant. Mean pairwise comparisons were computed with a Tukey’s test (α = 0.05). Means with the same letter are not significantly different and means with different letters are significantly different.

All analyses were performed with the R software version 3.4.2 (R Core Team, Vienna, Austria, 2017) [37].

## 4. Conclusions

1. The wear particles collected after wear corrosion in PBS and PBS-HA were mainly composed of chromium oxide coming from the detachment of the passive film and phosphate adsorbed on the particle surface and/or adsorbed on the broken passive film.

2. Composition of the corrosive medium and polarization, applied to mimic the electrical interactions observed in living tissues, has an influence on the chemical composition of the particles. The wear particles detached after wear corrosion with polarization in PBS-HA have a chemical composition with a higher significant content of Cr and Co than those particles collected without polarization.

3. Biocompatibility in vitro assays here reported, measured by LDH release and mitochondrial respiratory activity, seem to indicate that particles from wear corrosion in PBS supplemented with 3 g/L of hyaluronic acid, an approximate concentration that is found in the synovial fluid of healthy joints, under anodic polarization produce in macrophages lower damage to the plasma membrane and are more biocompatible, most likely associated with particles chemical composition.

4. As more variables of the prosthesis environment are considered in in vitro assays to study cell-biomaterial interactions, as are the electric interactions, in order to have a closer view of the different processes that are taking place in vivo at the cell-biomaterial interface, a better knowledge of the biological consequences will be obtained.

5. Understanding these consequences of the electrical signals on the growth and development of cells and tissues should be applicable for the design of appropriate solutions and adequate treatments for orthopedic-bearing patients.

## Figures and Tables

**Table 1:** Statistical analysis by Kruskal–Wallis test for the chemical composition (wt %) of wear particles detached during wear corrosion tests of CoCr samples in PBS (condition number 1), PBS+ 3 g/L HA (condition number 2) and under anodic potentiodynamic polarization (condition number 3), where *n* is the number of samples, mean is the average value, SD is the standard deviation, C25 is the value of the 25% of the data, C75 is the value of the 75% of the data, and *p* * is the significant difference at 0.95 confidence level.

	Condition Number	*n*	Mean	SD	Minimum	Maximum	C_25_	Median	C_75_	*p* *
Cr	1	7	33.28	6.92	21.61	43.13	29.33	34.38	38.45	
	2	24	28.53	8.73	14.06	48.82	21.28	27.52	33.66	0.034
	3	7	39.09	11.02	22.34	57.01	33.65	37.60	47.67	
Co	1	7	4.31	6.02	0.00	13.81	0.00	0.00	11.54	
	2	24	7.73	16.94	0.00	54.07	0.00	0.00	5.99	0.001
	3	7	36.08	17.59	15.14	58.64	17.57	32.12	51.48	
Mo	1	7	0.00	0.00	0.00	0.00	0.00	0.00	0.00	
	2	24	0.45	1.54	0.00	5.63	0.00	0.00	0.00	0.006
	3	7	2.71	2.60	0.00	5.48	0.00	4.00	5.45	
P	1	7	18.59	5.02	11.46	26.36	15.76	16.87	23.16	
	2	24	14.11	5.90	0.96	27.48	11.64	13.24	17.83	0.051
	3	7	9.40	7.30	1.30	19.57	1.89	8.79	16.10	
Al	1	7	1.52	4.01	0.00	10.62	0.00	0.00	0.00	
	2	24	0.18	0.68	0.00	3.17	0.00	0.00	0.00	0.123
	3	7	0.76	1.06	0.00	2.57	0.00	0.00	1.85	
O	1	7	40.10	10.67	23.79	54.36	33.72	41.31	51.57	
	2	24	41.82	17.72	3.52	72.84	34.64	49.54	51.69	0.002
	3	7	10.91	7.03	3.76	23.11	4.83	8.28	16.05	
C	1	7	2.19	5.80	0.00	15.35	0.00	0.00	0.00	
	2	24	6.98	7.49	0.00	19.94	0.00	6.39	14.16	0.022
	3	7	0.00	0.00	0.00	0.00	0.00	0.00	0.00	

* *p*-value in the Kruskal–Wallis test.

**Table 2:** Post hoc Mann–Whitney analysis to determine which pairs differed among them (condition numbers: 1-PBS, 2-PBS-HA, and 3-PBS-HA+POL).

	Comparison between Pairwise		
	*p* ** 1 vs. 2	*p* ** 1 vs. 3	*p* ** 2 vs. 3
Cr	0.119	0.277	0.021
Co	0.556	0.002	0.001
Mo	0.438	0.025	0.008
P	-	-	-
Al	-	-	-
O	0.508	0.002	0.002
C	0.098	0.317	0.017

** *p*-value in the Mann–Whitney test.

**Table 3:** Statistical analyses of cytotoxicity data. Mean differences of cytotoxicity effects between P2 (0.5 mg/mL) vs. P3 (0.5 mg/mL) and P3 (2 mg/mL) vs. P6 (2 mg/mL) were studied with Student’s *t* tests (α = 0.05), respectively. (**a**) Wear particles obtained in PBS (P2) and in PBS containing 3 g/L HA (P3). (**b**) Wear particles obtained in PBS containing 3 g/L HA (P3) and in PBS containing 3 g/L HA with polarization application (P6).

(**a**)		
P2 (0.5 mg/mL) vs. P3 (0.5 mg/mL)		
Mean of P2	Mean of P3	*P* Value
57.84	12.24	0.015
(**b**)		
P3 (2 mg/mL) vs. P6 (2 mg/mL)		
Mean of P3 (2 mg/mL)	Mean of P6 (2 mg/mL)	*P* Value
46.18	23.9	0.248

**Table 4:** Statistical analyses of mitochondrial respiratory activity. The effects of the particle (P), the concentration, and their interaction on the changes in respiratory activity were analyzed with a two-way analysis of variance. A *p* value of ≤0.05 was considered significant. Mean pairwise comparisons were computed with a Tukey’s test (α = 0.05). All analyses were performed with the R software version 3.4.2 (R Core Team, Vienna, Austria, 2017).

ANOVA				
	Sum Sq	Df	*F* Value	Pr (>F)
*P*	5745.2	1	266.75	4.64 × 10^−9^
conc	2191.39	2	50.87	2.76 × 10^−6^
*P* × conc	2648.33	2	61.48	1.07 × 10^−6^
Residuals	236.91	11	-	-

**Table 5:** As the interaction was significant, simple effects were compared. Means with the same letter are not significantly different.

P	Conc	Mean	Lower.CL	Upper.CL	Group
P3	2	25.7	17.1	34.3	a
P3	1	60.9	52.3	69.4	b
P3	0.5	87.5	77	98	c
P6	1	89.8	81.3	98.4	c
P6	2	94.3	85.8	102.9	c
P6	0.5	95	86.4	103.5	c

**Table 6:** Chemical composition (wt %) of High Carbon CoCr alloy (HCCoCr).

	C	Co	Cr	Mo	Ni	S	P	Al	W	Mn	Fe	Si	N	Ti	Cu
HC	0.22	62	29.4	6.4	0.1	0.004	0.001	0.01	0.03	0.7	0.16	0.7	0.16	-	-
